# Efficient service mesh traffic management for cloud-native applications

**DOI:** 10.1371/journal.pone.0344516

**Published:** 2026-03-12

**Authors:** Rizwan Ahmed, Shuyang Ren, Eunsam Kim, Choonhwa Lee

**Affiliations:** 1 Department of Computer Science, Hanyang University, Seoul, Korea; 2 School of Data Science and Artificial Intelligence, Wenzhou University of Technology, Wenzhou, China; 3 Department of Computer Engineering, Hongik University, Seoul, South Korea; Università di Pisa: Universita degli Studi di Pisa, ITALY

## Abstract

The cloud-native architecture and microservice technologies are revolutionizing the design, development, and management of cloud applications and services by offering greater elasticity, scalability, and flexibility. However, managing service-to-service traffic and handling faults turn out to be more difficult for modern, sophisticated cloud-native applications. The research community responded to the technical challenges by exploring efficient scheduling schemes that deploy constituent services to a node. Despite those efforts, current solutions are unable to handle real-time traffic dynamics, which could lead to resource waste and unnecessary communication delays. In this work, service partitions are used to improve resource distribution and traffic control in microservice-based applications. This strategy uses graph-based techniques to effectively cluster services, optimize resource usage, and boost communication efficiency, while continually monitoring application behaviors. We found that it can reduce response times by up to 15% during times of high network latency. The performance and dependability of microservices in cloud-native environments can be significantly improved using the proposed approach.

## 1 Introduction

Today’s software design and development advocate breaking down a large monolithic into smaller, loosely connected parts. Hundreds or even thousands of small services can be found in applications built with the microservice architectural style, each of which operates as a separate and cohesive process that communicates with its peers [1]. Using this approach, developers can work together on a single project using different platforms and programming languages, enabling independent component developments and upgrades with little disruption to the service the application provides. Additionally, operators can manage deployed services more easily.

A surge in input traffic for individual microservices can result in a violation of the Service Level Objective (SLO) of the application, because the performance of the application depends on individual microservices and their interaction triggered by the incoming request [2]. In such dynamic contexts, the inter-service communication and traffic control techniques are intricate. As a result, the orchestration of a constituent microservice set is made easier with the introduction of service mesh technology, which helped to tame the involved complexity [3].

In this work, we propose the use of application-level data to guide traffic routing decisions for distributed and containerized applications. Specifically, we continuously collect data relevant to the application context throughout the lifespan of deployed services, based on which we can model inter-service communications that reflect the internal interaction patterns of all components. More specifically, we use graphical representations of the interactions to create traffic scheduling decisions that minimize cross-service communication latency. The main motivation behind this approach is that by keeping cross-node communications as contained as possible, we can not only improve the application performance but also reduce the impact of unstable networks. However, two key challenges must be addressed to reach the goal: 1) determining which application-level data to collect and how to gather it and 2) formulating traffic scheduling decisions based on the information collected.

This paper is structured as follows. Section 2 provides the technical background on microservice technology, which serves as the foundation of cloud-native applications. Section 3 introduces our proposed architecture that features a service traffic steering scheme based on graph partitioning. The section elaborates on system design, including strategies for managing traffic uncertainty through predicted variance and an improved traffic management approach. Section 4 focuses on the evaluation and discussion of the proposed architecture, analyzing important performance metrics such as accuracy, fault tolerance, and reliability. Section 5 presents the existing research on traffic engineering and management for microservice-based cloud applications, identifying gaps in the existing literature. Finally, Section 6 concludes the paper by summarizing key contributions and discussing their implications for enhancing resilience in microservice architectures.

## 2 Microservice architecture technology

Microservice architecture refers to an architectural style in which applications are modeled as a set of small services with certain basic functionality [4]. More specifically, an application is modeled as a collection of loosely connected services that work together. Every service is expected to perform a specific function within the application. Various inter-service communication technologies exist with RESTful interactions being one of the most frequently utilized. A key advantage of adopting this pattern is the ease of implementing a request/reply protocol, while keeping it straightforward and eliminating the necessity for an intermediary broker of message queue protocols. The choice between using message queue-based or request/response-based interactions will hinge on the trade-off between the versatility and simplicity of the communication primitives in question. Each service must be crafted to enable independent development and deployment, leading to improved throughput and availability for the entire applications.

**Fig 1:**
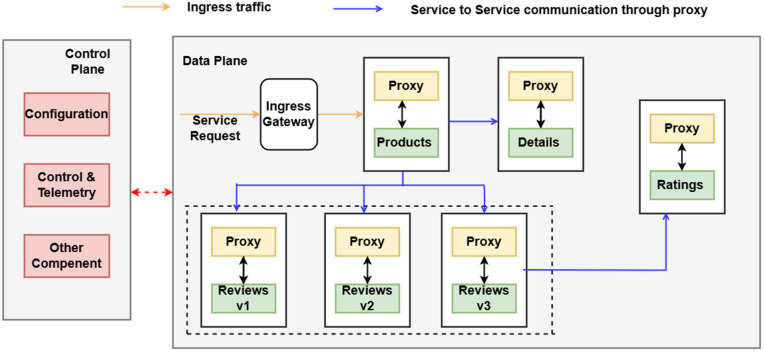
Bookinfo microservice-based application.

As a sample microservice application to present our proposal, we consider the Bookinfo application deployed on the service mesh platform [5]. It functions as an online book catalog that displays book details, descriptions, and reviews. As depicted in Fig 1, the application consists of Details, Ratings, and Reviews services that collectively present end-user data such as ratings and reviews. The product microservice serves as the front-end interface, utilizing its proxy to route requests to other services. Traffic management is handled by an internal load balancer that distributes requests across service instances registered in the service registry. This sample application is used as a guiding example throughout the paper.

### 2.1 Kubernetes platform

Kubernetes is a popular container orchestration platform that automates the deployment, scaling, and management of containerized applications. It serves as a foundation for the realization of microservice architecture by enabling efficient resource allocation, service discovery, and fault tolerance. With Kubernetes, companies and organizations can manage distributed workloads effectively and efficiently, ensuring high availability and scalability for cloud-native applications. Kubernetes auto-scaling feature refers to the capability by which resource allocations can be automatically adjusted in response to changes in application demand. To ensure that applications continue to function well under varying loads, the Horizontal Pod Auto-scaler (HPA) adjusts the number of pods in a deployment or replica set based on observable CPU, memory, or custom metrics. To maximize efficiency and usage, the Vertical Pod Auto-scaler (VPA) modifies the upper bound of resources allocated to each pod. Additionally, to preserve efficiency and save operating costs, the Cluster Auto-scaler (CA) dynamically grows a cluster’s node count based on upcoming workloads and resource demands. In dynamic environment, these Kubernetes auto-scalers improve the flexibility and resilience of cloud-native microservice applications by automating scaling.

### 2.2 Istio service mesh

As an intermediate layer between applications and underlying Kubernetes platform, service mesh layer abstracts application-related networking concerns from the applications themselves. In other words, it can be viewed as an infrastructural layer that makes it easier for microservices to communicate with each other. Without requiring modifications to the application code, it offers vital features like fault tolerance, traffic management, load balancing, service discovery, and security. Because it guarantees persistent and dependable inter-service communications even in changing network conditions, service mesh technology is especially useful in cloud-native computing environments where applications are deployed as microservices.

A handful of implementations of the technology can be found at https://layer5.io/serivce-mesh-landscape, which include Istio, Linkerd, Consul, NGINX Service Mesh, and so on. Istio is one of the most popular service mesh technologies that provides a comprehensive solution to meet the needs of application-specific networking functionalities for microservice applications. An envoy proxy is set up as a sidecar to its corresponding service container that takes care of all the application-level networking details. Istio offers microservices sophisticated security, observability, and traffic management. By supporting a rich set of advanced capabilities such as request routing, retries, circuit breaking, and mutual TLS authentication, Istio provides developers with fine-grained control over inter-service traffic and security policies without concerns affecting application code, so they can concentrate on business logic. More specifically, the technology provides various features to manage service traffic, such as request timeout and retries, rate limiting, and circuit breaking, to make applications more resilient against network instability and dependency on other services. Rate limiting works by rejecting incoming requests to protect downstream services in the case of an excessive input surge. It allows faster responses to traffic spikes or overloads than traditional methods like auto-scaling [6]. The retry setting controls how many times a service will try to connect to another service, if the current attempt fails. Also, the timeout between successive retries helps prevent the called service from getting overwhelmed by too many requests. Rate-limiting is applied to important services to prevent them from being overloaded with excessive requests.

### 2.3 Distributed service tracing and observability

Resiliency, fault tolerance, and service efficiency are important goals for any technically mature distributed service platform. The first step towards them should be the observability of distributed cloud-native applications that will open up the gate for understanding individual service behaviors and their interactions. The observability of distributed networking applications can be collectively enabled by a set of event collection and aggregation, distributed tracing, and analytics technologies. We provide an overview of outstanding building blocks and tools in this arena, which includes Prometheus, Grafana, and Loki.

**Prometheus:** An open-source monitoring and alerting package called Prometheus was created with scalability and dependability in mind for cloud-native settings. In addition to providing a robust query language (PromQL) for analysis, it gathers real-time metrics from applications and infrastructure elements and saves them in a time-series database. Prometheus is frequently used to watch resource usage, keep an eye on system performance, and send out alerts according to pre-set thresholds.

**Grafana:** It is a platform for analytics and data visualization that creates dynamic dashboards by integrating with Prometheus and other data sources. It enables users to track the health of microservices and infrastructure in real time, visualize metrics, and identify anomalies. Grafana is a desired option for monitoring large dispersed applications and systems because of its adaptable panels and alerting features.

**Loki:** The highly accessible, horizontally scalable log aggregation system, Loki, was created with Grafana integration and efficiency in consideration. In contrast to conventional log management systems, Loki simply indexes metadata, not the entire log content, allowing for faster queries and more affordable storage. Loki offers a comprehensive observability solution when paired with Grafana, enabling developers to correlate logs with metrics and learn more about the behavior of the system in question.

The service resiliency can be extended beyond the cluster boundary, which is called multi-cluster failover capabilities; service traffic is automatically re-routed to an available service replica in another cluster, if a service instance becomes unavailable. The usual method to achieve this functionality is through health checks. The service mesh re-routes traffic to a healthy replica, when a service is flagged as unhealthy. Examples of this implementation include AWS App Mesh [7], Traffic Director [8], Linkerds failover extension [9], and Istios locality load balancing [10].

## 3 Intelligent microservice traffic management

To address the growing need for adaptive and intelligent traffic control in microservice-based cloud-native systems, our work introduces a graph-based dynamic routing solution that enhances system performance, fault tolerance, and scalability. The primary objective of our design is to ensure optimal traffic allocation across microservices, taking into account real-time resource utilization and communication constraints, while minimizing latency and maximizing service quality.

### 3.1 System design principle

Microservice applications are modeled as a directed acyclic graph, based on which intelligent routing decisions are made. Our proposed architecture offers resource usage and end-to-end traffic optimization through Microservice Communication Graph (MCG)-based method. This method intelligently reroutes incoming requests to alternate service instances with lower traffic consumption, ensuring improved usability and dynamically handling microservice failures. Through resource optimization and service continuity, the graph-based approach can improve system scalability, robustness, and user experience. We first present a set of design principles that are at the center of our traffic steering system design.

**Fine-grained traffic routing via graph modeling:** To enable intelligent routing decisions, we adopt the Microservice Communication Graph (MCG) model [11]. This model abstracts each microservice as a node and each communication path as a directed, weighted edge. Edge weights are determined by telemetry metrics such as response time, request rate, or traffic intensity, while node properties reflect real-time CPU and memory usage collected from Prometheus system. This allows for dynamic adaptation to ever-changing service demand and system conditions.

**Real-time monitoring with low overhead:** We utilize Prometheus as a lightweight monitoring back-end to continuously collect metrics such as service’s CPU/memory usage, number of requests per second, and latency. This monitoring does not require service instrumentation and relies on sidecar proxies and Kubernetes-level metrics, reducing operational overhead.

**Optimized resource utilization and fault tolerance:** Our approach not only ensures a balanced load distribution across replicas, but also proactively addresses service disruptions. When performance metrics surpass defined thresholds, e.g., more than 50 requests in 30 seconds, additional replicas are dynamically spawned to handle the increase. These replicas are then integrated into the routing strategy to prevent bottlenecks, conserve bandwidth, and enhance fault tolerance.

**Fig 2:**
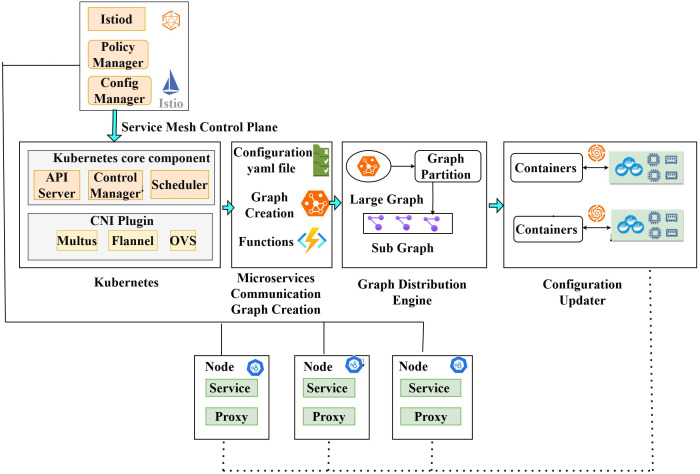
Intelligent service and traffic management system architecture.

As illustrated in Fig 2, the proposed system comprises three core modules: MCG(Microservice Communication Graph) Generator, Graph Distribution Engine, and Configuration Updater. The MCG Generator interacts with the Kubernetes control plane and sidecar proxies to build and maintain an up-to-date snapshot of inter-service communication patterns. The service graph topology is periodically sent to the Graph Distribution Engine, which partitions and distributes the graph partitions based on load-balancing and traffic co-location constraints for the scheduling. The Configuration Updater then converts the graph partitions into YAML-based traffic scheduling constructs such as circuit breaking, retries, and replica management, and applies them via the orchestrator.

**Fig 3:**
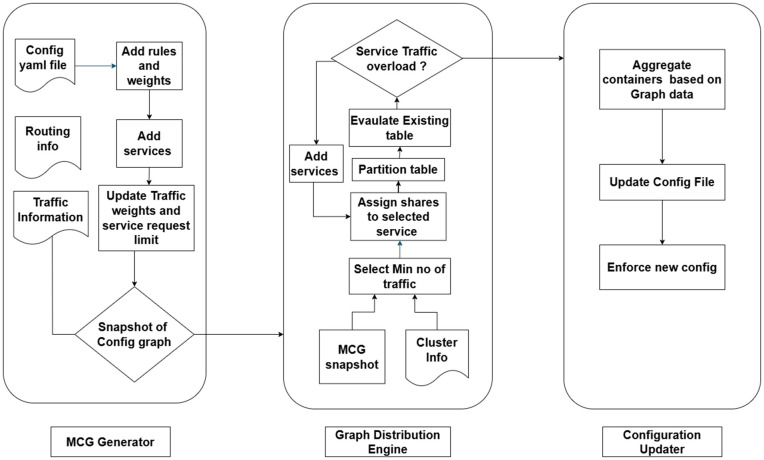
Flow of dynamic scheduling decisions for traffic management and resilience.

Fig 3 sketches the workflow of our solution. Our proposed system dynamically configures scheduling decisions in the distributed environment to ensure optimal performance. There are three YAML files: Config, Routing, and Traffic Information. In the Config file, it defines rules such as connection pool settings for TCP and HTTP, specifying the maximum number of TCP connections, HTTP max pending requests, and maximum requests per connection. When certain services fail or become unavailable, rules can be included to define request delays and abort requests. The Routing Information YAML file contains service images along with their maximum and minimum availability. For instance, 60% of the traffic is directed to services with a five second delay, while 40% of the traffic is routed to other services with a six second delay, and 10% of requests fail with an HTTP 500 server error. Finally, in the Traffic Information file, it adds snapshots of the graph scheme [11] to generate traffic information, route the traffic, dynamically update the configuration, and adjust the weights accordingly.

### 3.2 MCG generator

Sidecar proxies, which are added to each microservice at the start, are used to route request messages among service instances. Also, they are used to collect numerous types of metrics. The MCG generator regularly checks these metrics related to inter-service communication. For example, Envoy sidecar proxies collect standard metrics and labels for TCP, HTTP, and gRPC traffic and send these data to the Prometheus time series database [12]. Since we are interested in response time and how much traffic is passing through, we primarily look at the request count and how long each request takes. Another option for building a service graph is to use Kiali [13], a tool that helps manage and visualize the service mesh. However, using Kiali adds extra software components and may slow things down. After collecting the data, we create a graph to organize the information. Let G=(V,E) be a graph where:

- Vertices V are application instances extracted from deployment manifests.
- The weight of a vertex $v_{i}$ is the service requirement, e.g., services replica, request limit, and pending requests from users.
- An edge (u,v) is created, if vertex u communicates with vertex v.
- The weight of an edge (u,v) is denoted with the vector ⟨λ(t),r(t)⟩, where λ(t) and r(t) are the traffic intensity and response time collected from the data plane.

While vertices and their weights in a service DAG remain static once instantiated, the edges and edge weights may change over time depending on the incoming requests. Therefore, the creation of a service graph is not a static process. We continuously collect communication information and update the service DAG throughout the lifetime of an application. We take a snapshot of the most recent graph based on a configurable timetable T. The edge weights are also updated using a moving average of the same time window to filter out noise and bursts.

**Algorithm 1.** Construction of the Microservice Communication Graph (MCG)

**Input:** *S* Set of microservices deployed in Kubernetes

**Input:** *T* Time window for snapshot aggregation

**Output:** G=(V,E) Microservice Communication Graph

 1. Initialize vertex set V←∅ and edge set E←∅.

 2. For each microservice s∈S:

   • Add *s* to vertex set *V*.

   • Retrieve metadata such as replica count, request limits, and pending requests.

   • Assign this information as the vertex weight w(s).

 3. For each communicating pair $(s_{i},s_{j})\in S$:

   • Monitor traffic metrics between $s_{i}$ and $s_{j}$ over time window *T*.

   • Collect metrics: λ(t) (traffic intensity) and r(t) (response time).

   • Compute moving averages $\bar{\lambda }$ and $\bar{r}$ over *T*.

   • Create or update edge $\left(s_{i},s_{j}\right)$ with weight $\langle \bar{\lambda },\bar{r}\rangle$.

   • Add edge $\left(s_{i},s_{j}\right)$ to edge set *E*.

 4. Return G=(V,E).

As shown in Algorithm 1, the MCG Generator is responsible for dynamically building a communication graph that accurately reflects the current interaction patterns among microservices within the Kubernetes environment. We provide a formal description of the graph partitioning-based algorithm to clearly delineate the proposed microservice deployment strategy. The approach uses a weighted graph to describe inter service communication, with nodes standing in for microservices and edges for communication costs. While preserving load balance among nodes, the partitioning attempts to reduce inter-partition latency. This algorithm is executed by the MCG Generator component of our system and leverages telemetry data collected from the service mesh data plane, more specifically from Envoy proxies through Prometheus. The algorithm begins by scanning the list of all deployed microservices and instantiates a node in the graph for each service. Each vertex is annotated with service-specific metrics, such as its resource usage and incoming request rate, which act as the node weights. Following the vertex creation, the algorithm then evaluates all service pairs to determine whether a communication link exists between them. If communication is detected, it adds a directed edge to the graph representing this connection. Each edge is annotated with a weight vector that includes values such as traffic intensity requests per second, response time, and pending request queues metrics that are vital for understanding service dependency and performance bottlenecks. This graph evolves over time, and the algorithm includes a periodic update mechanism using a moving average technique to smooth sudden metric spikes, while being kept up-to-date with recent interactions among microservices. The result is a real-time, weighted directed graph that represents both service topology and traffic characteristics, forming the foundation for intelligent traffic routing and optimized deployment decisions.

**Fig 4:**
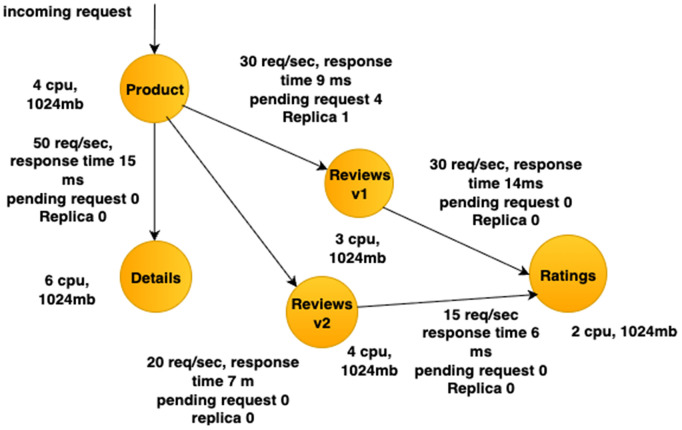
Service graph of Bookinfo application.

Fig 4 shows a DAG service topology of the BookInfo application [5] generated by the MCG based on real-time traffic and communication data. The graph depicts how the microservices in the application are interconnected together. Each node in the graph represents a microservice, annotated with CPU and memory usage to reflect resource usage. The edges between services indicate active communication links with weights showing the number of requests per second and average response time. Using Algorithm 1, this graph is dynamically constructed from telemetry data to capture current service interactions. This graph is then processed by Algorithm 2 to generate optimal traffic routing and deployment configurations that ensure low latency, fault tolerance, and efficient resource utilization.

### 3.3 Graph distribution and scheduling

We represent the co-location scheduling as a graph distribution and mapping problem using a Microservice Communication Graph (MCG) snapshot.This generates a set of subgraphs which are then assigned to cluster nodes for deployment.The scheduling decisions are guided by two main conditions: the first is that each nodes allocated subgraph must satisfy the resource requirements of its microservices, represented by the vertex weights, and the second is that the overall inter-node communication latency, represented by the edge weights, must be minimized.

The reason for modeling it as a graph scheduling problem instead of a standard bin packing problem is that the second condition is equal to the main goal of the graph distribution problem, which is to minimize the (weight of) edge cuts. Furthermore, we use unequal-size graph partitioning, one of the alternatives offered by the current graph distributing tools to meet the first condition [14]. Lastly, a solid foundation and hidden graph data are already provided by the graph-based representation of service communications, which easily corresponds to the graph-distributed formulation without any changes

The edge weight in an MCG graph takes into account response time, pending requests, and traffic intensity. When it comes to graph distribution, we have the option to utilize both or either of these factors, and the resulting distribution outcomes may vary based on the specific application. Opting to minimize response time suggests a preference for grouping components with heavier interactions leading to a higher likelihood of being placed on the same node with lighter traffic intensity being situated on different nodes. While this can enhance long-tail latency, it might also lead to an increase in average response time. Conversely, minimizing traffic intensity indicates a desire to group components that communicate more frequently, which can be particularly advantageous for services that prioritize throughput.

The service availability of all cluster nodes is gathered by the Graph Distribution Engine from the container platform to determine the ranking of the cluster nodes. Based on this information, top N cluster nodes with sufficient resources to accommodate the services are selected. This selection is based on the assumption that consolidating service workload onto fewer cluster nodes is more preferable rather than distributing the workload evenly. Following this, the ratio of available services of the N-selected cluster nodes is computed, and it is used as the target ratio for the unequal-size graph distribution. The ratio and the MCG snapshot are then utilized as input for the graph distribution tool. Although our prototype currently uses Pandas [15], it can be substituted with other graph distribution tools.

**Algorithm 2.** Dynamic Configuration Generation using MCG

**Input:** G(V,E) Microservice Communication Graph

**Input:** *P* Traffic steering decisions

**Input:** *T* Monitoring window

**Input:** *DB* Metrics database

 1. For each vertex v∈V:

   • Collect CPU and memory usage from *DB* over the window *T*.

   • Set resource configuration for *v* based on usage trends.

 2. For each edge (u,v)∈E:

   • Collect λ(t) and r(t) from *DB* over the window *T*.

   • Compute weighted average ⟨λ(t),r(t)⟩.

   • If thresholds defined in *P* are exceeded: if the performance thresholds defined in P (such as latency or error-rate limits) are exceeded, apply circuit breaking, or retry policies in the configuration

    – Apply circuit breaking or retry policy in the configuration.

 3. Generate configuration YAML with:

   • Routing information (percent splits, failover)

   • Connection pool settings

   • Retry and timeout rules

 4. Deploy the generated configuration using the Configuration Updater.

As shown in Algorithm 2, the process of generating dynamic configuration files using the MCG generator operates on a graph where microservices are modeled as nodes and their communication links as edges. For each microservice node, the system retrieves CPU and memory utilization data from the monitoring database aggregated from Prometheus over a defined time window. Based on these usage patterns, the system configures appropriate resource requests and limits, ensuring optimal resource allocation. Also, for every communication link in the service graph, the system collects telemetry data such as the number of requests per second (*λ*) and the average response time (r). These metrics are used to compute a weighted average that reflects service interaction behavior during the monitoring window. If the computed values exceed predefined thresholds specified in the traffic policy rules such as high latency, excessive request load, or insufficient resource availability, the algorithm triggers appropriate configuration responses like circuit breaking, retry scheduling decisions, or timeout adjustments. In addition to these basic scheduling decisions, the system applies adaptive traffic scaling strategies. For example, if a microservice is observed to handle more than a particular number of requests, the algorithm evaluates the current bandwidth and CPU thresholds. If system capacity allows, it deploys an additional replica of the overloaded microservice, enabling traffic to be redirected or load-balanced across multiple instances. This duplication helps maintain service stability and responsiveness without breaching system limits. Finally, after analyzing both service-level and communication-level metrics, the algorithm compiles the results into a configuration file in YAML format. This file includes detailed service routing rules, load balancing strategies, retry and timeout configurations, and connection pool settings. The finalized configuration is passed to the Configuration Updater component that deploys it to the service mesh via the service orchestrator, enabling fine-grained, real-time traffic control and fault tolerance based on actual communication behaviors within the service network.

**Algorithm 3.** Microservice Placement based on Graph Partitioning

**Input:** G(V,E) Microservice Communication Graph

**Input:** *R* Available resources across nodes (CPU, memory, bandwidth)

**Input:** *C* Communication cost between nodes

**Input:** *W* Workload demand vector for each service

**Output:** *M* Placement mapping of microservices to nodes

 1. Initialize empty mapping M={}.

 2. Compute edge weights w(u,v) for each (u,v)∈E based on communication frequency or latency.

 3. Apply graph partitioning on G(V,E) to minimize inter-partition communication cost:

   • Use a partitioning function Partition(G,k) that divides *G* into *k* balanced subsets, ensuring $\sum_{v_{i}\in V_{p}}W(v_{i})\leq R(p)$ for each node partition *p*.

 4. For each partition *p* obtained:

   • Select a node *n* with sufficient resources in *R*.

   • Assign all microservices in partition *p* to node *n*.

   • Update mapping M[v]←n for all $v\in V_{p}$.

 5. Optimize placement iteratively:

   • For each (u,v)∈E, if *u* and *v* are placed on different nodes and communication latency >τ:

     – Migrate service *v* to node of *u* if resource capacity allows.

     – Update mapping *M* accordingly.

 6. Return final placement mapping *M*.

The microservice placement procedure based on graph partitioning is explained in Algorithm 3. In the communication graph G(V,E), each microservice is represented as a vertex, and the edges show the relationships between the services in terms of communication. In order to determine communication latency or intensity, the method first calculates edge weights. The graph is then divided into balanced subsets to reduce the cost of inter-partition communication, while maintaining respect for each node’s resource limitations. Every partition is mapped to a particular node that has enough resources, and when communication latency between dependent services grows beyond a predetermined threshold, the placement is incrementally improved by transferring services. This procedure guarantees better tightly connected microservice co-location, which lowers inter-node latency and improves system performance.

### 3.4 Configuration update

Configuration Updater is a platform-dependent component. It converts the graph data from the Graph Distribution Engine to executable configuration scripts/templates and enforces them through the container platform orchestrator. There can be different ways to implement this component depending on the container platform. For example, for Kubernetes orchestration platform, we can integrate this component with the control plane by extending the default scheduler. The other option is to keep this component agnostic of the underlying platform and to define a deployment manifest file in terms of existing node/pod affinity and anti-affinity rules. Our current implementation uses the second approach. Note that, in our prototype, it takes a while to collect enough metric information to create an MCG snapshot graph. Therefore, it relies on the default traffic scheduling decisions to make the initial traffic decisions.

The dynamic configuration mechanism in our system is responsible for generating and applying various YAML configuration files based on metrics collected via the MCG component. These YAML files reflect adaptive traffic control, resource optimization, and fault resilience strategies tailored to the actual behavior of microservices.

**Fig 5:**
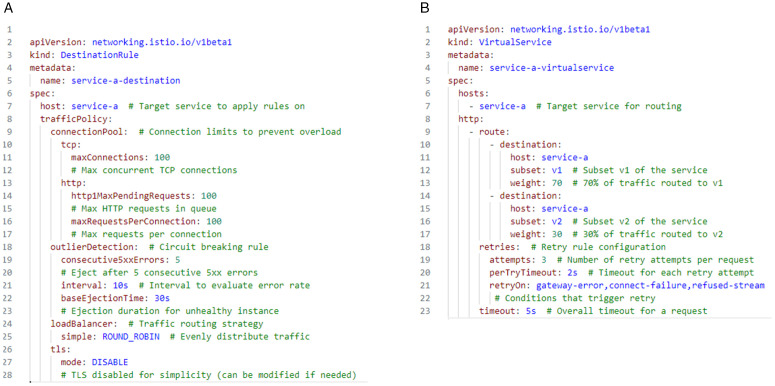
Dynamic configuration rules for microservices. **(a)** Circuit breaking and load balancing. **(b)** Retry, timeout, and traffic splitting.

Fig 5 defines the dynamic configuration process enabled by the MCG scheme. The YAML file in Fig 5 (a) specifies circuit breaking and traffic routing rules for a microservice using a DestinationRule object. It includes performance-relevant controls such as connection pool limits, circuit-breaking rules through outlier detection, and a load balancing strategy of round-robin routing. For instance, the configuration limits the number of concurrent TCP connections and HTTP pending requests to prevent service overload. Additionally, unhealthy instances that return five consecutive 500 errors are automatically ejected for a defined interval, helping ensure fault tolerance. These rule values can be dynamically adjusted based on historical telemetry data (e.g., service response times and failure rates) to proactively avoid performance degradation.

The second YAML file in Fig 5 defines retry, timeout, and traffic splitting rules using a VirtualService object. It enables adaptive traffic distribution between different subsets (versions) of a microservice, e.g., routing 70% of traffic to version v1 and 30% to v2. This supports dynamic scaling and traffic shifting decisions. When the system detects sustained request rates above a threshold (e.g., more than 50 requests within 30 seconds) and the resource utilization (CPU, memory) remains within limits, a new replica of the service may be deployed. The Configuration Updater then adjusts routing weights to direct a portion of the traffic to the new replica. Furthermore, retry rules (e.g., 3 attempts with a 2-second timeout) and an overall request timeout of 5 seconds are defined to improve system resilience against network delays or transient failures.

## 4 Experimental evaluation

We conducted a set of experiments, using a prototype implementation of the proposed approach which is made publicly available [16]. The cluster machine runs Ubuntu 18.04 LTS, configured with Docker 20.10.7, Kubernetes 1.20.0, and Istio 1.8.2. The cluster consists of one control plane node and two worker nodes each allocated 4 CPU cores and 8 GB of memory. Our proposed architecture implements a dynamic graph-based scheduling scheme for traffic management, offering a more adaptive and efficient approach compared to the default traffic management scheduling decisions provided by Istio. The comparison demonstrates that our proposed method significantly improves performance and flexibility in handling diverse traffic patterns.

### 4.1 Service response time

The Bookinfo application [5] was employed in our experiments. The application is made up of four microservices of a web product page, book details, reviews, and ratings. Istio was utilized to demonstrate the core phases of traffic management for the services. For the experiments, we created two separate instances of the Bookinfo application in different Kubernetes namespaces within the same service mesh. The first instance implemented MCG’s dynamic scheduling decisions, whereas the second used Istios default control plane. A global configuration service also provides essential configuration data for all services, including service discovery, network routing rules, and network security scheduling decisions.

This experiment evaluates how adaptive microservice placement affects latency under varying network delays. A series of performance evaluations using Locust, a distributed load testing tool [17], to simulate realistic traffic patterns in the Bookinfo microservice-based architecture deployed with a service mesh and our proposed MCG-based microservice placement scheme. The tests involved sending HTTP requests to test API endpoints such as /product/test, /reviews/test,/details/test, and /ratings/test, with variations in both request volume and payload size. Payloads ranged from lightweight JSON bodies (1–2 KB) to more complex structures (10 – 15 KB). Under moderate load conditions (100–300 requests per second), both the default service mesh and the MCG approach performed comparably, with low response times and stable communication. However, as traffic intensity exceeded 500 requests per second with maximum payloads, the service mesh-only setup exhibited noticeable degradation due to the cumulative overhead introduced by sidecar proxies, telemetry collection, and routing logic. In contrast, the MCG placement strategy, by co-locating heavily communicating services and minimizing inter-node traffic, mitigated much of this overhead, leading to lower response times and improved system stability.

**Fig 6:**
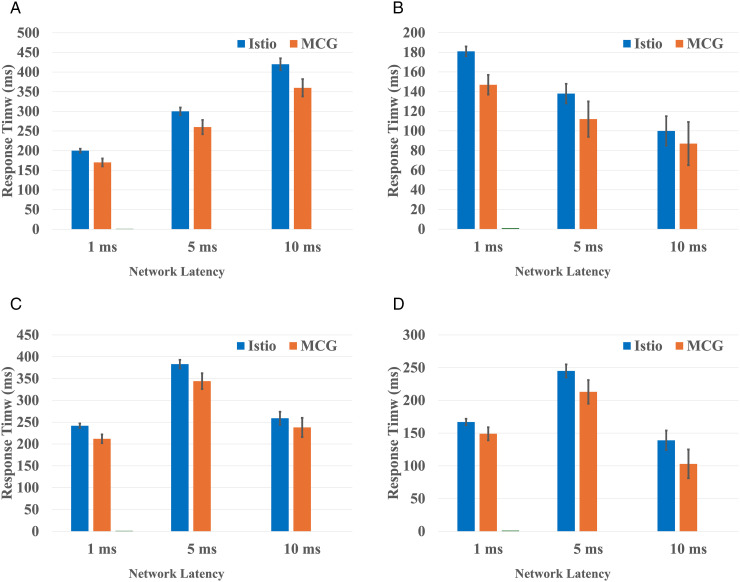
Average response time of Bookinfo microservices. **(a)** Product service. **(b)** Ratings service. **(c)** Details service. **(d)** Reviews service.

Fig 6 plots the variation in the average response time of each microservice under three network latency conditions (1, 5, and 10 ms), as the input request rate increased from 985 to 998 requests per second. The x-axis represents the configured latency between services, while the y-axis denotes the average response time in milliseconds measured during steady state operation. Error bars indicate the standard deviation observed across multiple experimental runs, reflecting the variability and stability of each services performance under fluctuating load conditions.

The Reviews service comprises two versions (v1 and v2), but since their average response times were nearly identical, we present a single aggregated value to simplify interpretation. The default service mesh configuration struggled to adapt when network latency dropped unexpectedly, e.g., from 5 ms to below 1 ms, leading to premature throttling and inconsistent service states due to static rate-limit and health-check parameters. In contrast, the MCG-enabled deployment maintained stable performance, adapting more effectively to rapid latency variations.

Under peak load conditions (800–1000 requests per second with large payloads), the service mesh-only configuration experienced HTTP 500 errors, especially in the Ratings service, due to timeouts and resource saturation. The MCG-enhanced setup demonstrated better resilience, with fewer failed requests and more predictable behavior. These findings confirm that integrating intelligent traffic management strategies such as MCG can improve system robustness, reduce latency, and sustain throughput under dynamic network conditions.

The results showed that the MCG approach strengthens the system’s performance with respect to flow response time and network stability. The MCG placement strategy outperforms the Istio default control plane, achieving about 10–15% drops in the response time in normal network conditions. Markedly, in increased network latency, the approach improved the response time by 15%, which can be attributed to the fact that the service co-location strategy to reduce inter-service communication delay. The co-location placement scheme mitigates the negative performance, and its impact is linked to the network delays, guaranteeing a more stable response time.

### 4.2 Circuit breaking and request failures

Another set of experiments utilizes *μ*Bench benchmarking tool that is designed to evaluate the performance of microservice-based applications [17]. It generates a synthetic application composed of dummy microservices that can be deployed on a Kubernetes cluster. Each microservice in the application is categorized into three distinct types of traffic: internal bytes, external bytes, and return bytes. The internal services are primarily responsible for stressing specific system resources such as CPU, disk, or memory, and for generating artificial network traffic to simulate real-world load conditions. The external services represent inter-service communication, simulating the interactions between microservices across the application. In contrast, the return bytes refer to the dummy data sent back to the caller by the internal service after completing its request.

Benchmarking task follows mainly three steps: Kubernetes Deployer, Work Model Generator, and Service Mesh Generator [17]. The Service Mesh Generator generates a random service mesh for a microservice application. A service mesh is a collection of external services that are called by each service. The services represents the nodes in a service graph. Configurations are imported by the Work Model Generator module from a servicemesh.json file. Automatically generated by the Service Mesh Generator module, this file includes service mesh parameters that are mostly based on the needs of the application. We use the Kubernetes Deployer to deploy all the services.

#### 4.2.1 Default behavior.

The evaluation was performed using the *μ*Bench benchmark without enabling the Microservice Communication Graph (MCG) scheme without any kind of traffic allocation, dynamic routing, or fault-handling features. The purpose was to observe the default system behavior under stress and failure conditions. The experiment represented a high-load scenario with 290 concurrent users, spawning at a rate of 100 users per second, and sustaining for a duration of 300 seconds. To evaluate fault tolerance, two types of controlled faults were injected during the test period. The first 3-second delay was introduced to 50% of the requests targeting a *μ*Bench service, simulating backend slowness or network latency. The second 20% of requests to a dependent downstream service were forcibly terminated using HTTP 500 Internal Server Errors, emulating service crashes or unhandled exceptions. No circuit-breaking, fallback, or intelligent rerouting mechanisms were present to mitigate these faults. As a result, all user requests were directly forwarded to services without isolation or error handling, exposing the system to the full impact of the faults.

**Fig 7:**
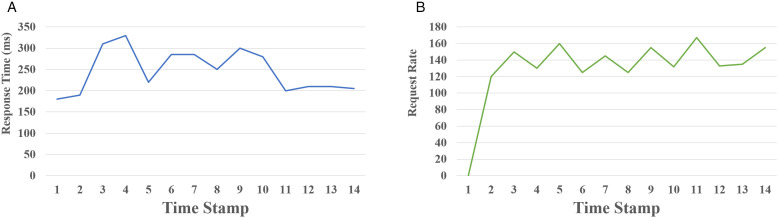
Behavior of no traffic management. **(a)** End-to-end latency. **(b)** Request rate.

As presented in Fig 7, the systems behavior without MCG or fault isolation mechanism is shown under fault injection conditions. The x-axis in both plots represents timestamps at 10-second intervals. In Fig 7 (a), the end-to-end response time ranges between 165 and 337 milliseconds, with noticeable spikes during periods of fault injection, reflecting sensitivity to service-level disruptions. In Fig 7 (b), the total number of requests per second fluctuates between 120 and 167 rps, indicating an unstable throughput pattern. This inconsistency emphasizes that the system faltered to maintain a steady load under failure conditions, with no assurance of successful request handling or satisfactory user-level performance. In the absence of MCGs fault isolation and placement mechanisms, the system lacked resilience. Several failed requests propagated errors to upstream services, amplifying instability. The lack of fallback mechanisms or timeout handling resulted in direct performance degradation. These results highlight the need for a dedicated communication and traffic management scheme.

#### 4.2.2 MCG-enabled case.

To evaluate the system’s resilience under fault conditions, circuit breaking mechanisms were applied alongside the MCG scheme, using service mesh features. In this experiment, we deliberately injected faults into microservices by introducing a 30% error rate (HTTP 500 responses) and adding a 500 ms delay to 20% of the traffic, simulating partial slowness and failures in downstream services. To contain service disruptions, Istios circuit breaker was configured; the thresholds included a maximum of 100 concurrent connections per host, a connection pool size of 50, and outlier detection parameters where a service instance would be ejected after 5 consecutive errors. Once ejected, the instance remained out of rotation for 10 seconds before being re-evaluated for reentry. These conditions ensure that repeatedly failing instances were promptly isolated, thereby minimizing the risk of system-wide degradation.

**Fig 8:**
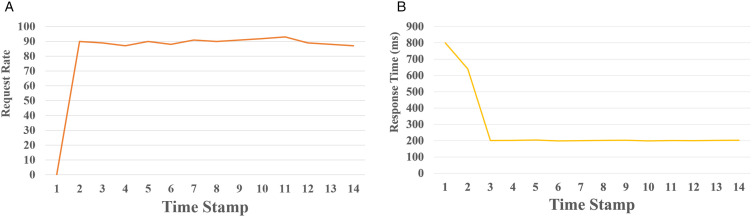
Performance under MCG-enabled scenario. **(a)** End-to-end response time. **(b)** Request rate.

Fig 8 demonstrates the system’s improved stability and responsiveness under the MCG scheme with Istio-enabled circuit breaking. Fig 8 (a) illustrates a significant drop in the 95th percentile response time from 800 ms to around 200 ms, reflecting the systems ability to recover from initial performance degradation and stabilize quickly. Each timestamp in the graph represents a 10 second interval. Fig 8 (b) shows that the system sustained a stable throughput of approximately 90 requests per second after an initial warm-up phase, indicating balanced request distribution under normal operating conditions. These results were obtained using Locust to simulate a cold-start scenario followed by sustained load. During the initial phase when there is no traffic, the system records 0 RPS but exhibits a high response time of 800 ms, indicating startup delays possibly due to container boot time or service initialization. From timestamp 2 onward, as traffic gradually ramps up to 90 RPS, the system quickly stabilizes. The response time drops sharply to approximately 200 ms by timestamp 3 and remains consistent through timestamp 14, despite minor fluctuations in RPS.

**Fig 9:**
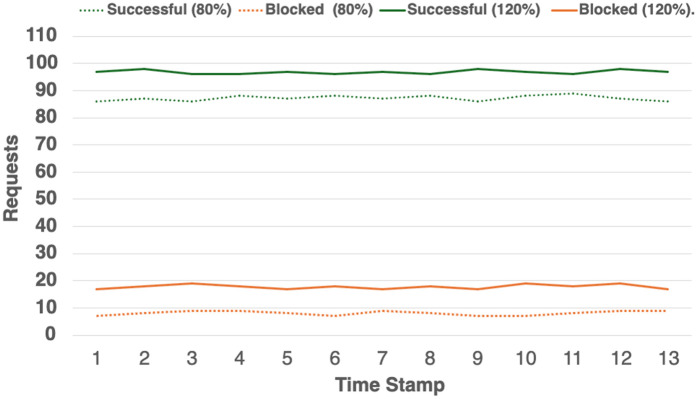
Impact of varying inbound traffic load.

To investigate the impact of varying incoming traffic volume, we conducted a set of experiments in which the emulated workload was varied between 80% to 120% of the capacity. In the experiments, we limited the maximum request queue length to 20. The status code frequencies observed under these conditions are shown in Fig 9. It is evident that the volume of incoming traffic directly influenced both the maximum request rate and the response time, depending on the broken circuit’s maximum queue length. A large queue length allows more requests to pass through, thereby increasing the request rate, but this also results in higher response times. Conversely, a smaller queue length reduces response times, but also limits the request rate. Moreover, the volume of incoming traffic affected the frequency of unsuccessful requests. Although the rate of successful requests was higher in Fig 9, when the traffic volume reached 120% of capacity, this led to an increase in the frequency of unsuccessful requests in the *μ*Bench microservice-based architecture. In short, the results show that enabling fast failures helped the application maintain an acceptable response time under high traffic conditions.

**Fig 10:**
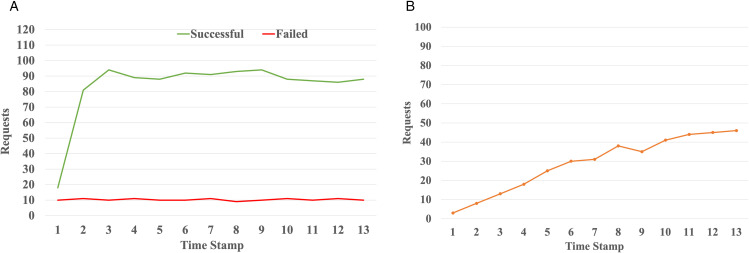
Impact of workload spike and circuit breaker. **(a)** Successful and failed requests. **(b)** Blocked requests.

Fig 10 shows the effect of the request timeout and retry mechanism under conditions of persistent overload. Retries are commonly used to handle input spikes or transient failures. To study the impact of workload spikes and consequent retries, we conducted experiments where the incoming traffic was set to 100% of capacity under normal conditions, spiking for 5 seconds. In the experiments, the requests queue length was set to 20, and the number of permitted retry attempts was set to 10. We used a retry interval of 5 seconds throughout the experiments. In Fig 10 (a), successful and failed requests are indicated by the green and red lines. The number of incoming requests reached approximately 120 requests per second with around 97 successful RPS on average, indicating some capacity loss due to broken circuits. Fig 10 (b) plots the number of retries triggering request blocking. With a retry interval of 5 seconds, the impact of the input spike is mitigated. As a result, the retries are more evenly spaced as shown in the graph, improving recovery stability. In other words, the system experienced fewer occurrences of circuit-breaker activation than a short retry interval. When the retry interval is set to 2 seconds, request retries are triggered too frequently, leading to too many repeated requests.

The results demonstrate that the MCG placement strategy plays a vital role in reducing latency, ensuring system resilience, and stable performance even under high load conditions. Frequently communicating services are co-located by the MCG scheme to achieve such performance improvements. These results support previously researched literature on the importance of fault tolerance and traffic co-location in microservice-based systems [15]. In other words, proper arrangement of circuit breakers, service placement, and retry strategies can improve system dependability, even when the network delay and traffic spike are present.

#### 4.2.3 Threats to validity.

We discuss the threats to validity of our experimental results, following the commonly used classification into internal, construct, external, and conclusion validity.

**Internal validity:** All experiments were conducted in a controlled Kubernetes cluster where failures were injected under predefined configurations (e.g., fault types, injection timing, and network conditions). Despite our efforts to keep deployments uniform, the outcomes may still be influenced by uncontrolled runtime factors such as non-deterministic pod scheduling decisions, background resource contention on cluster nodes, and transient effects introduced by autoscaling. Moreover, fault injection itself can introduce timing variations (e.g., failures occurring during load spikes versus steady state), which may lead to different levels of service degradation. To reduce these effects, we repeat each experiment multiple times under identical configurations, warm up the system before measurement, and report averaged metrics. Nevertheless, some residual variability may remain due to the inherently dynamic nature of containerized environments.

**Construct validity:** We evaluate the effectiveness of the proposed deployment approach using throughput, end-to-end latency, and stability indicators, which are widely adopted in microservice benchmarking and cloud performance evaluation. However, these metrics may not fully capture all dimensions of microservice behavior and operational cost. For instance, our evaluation does not explicitly quantify inter-service communication overhead (e.g., queueing delays between services), tail latency under rare events, or resource efficiency aspects such as CPU efficiency and energy consumption. Additionally, the interpretation of some metrics can be affected by middleware configuration, including service mesh behavior (e.g., Istio retries, timeouts, and circuit breaking), load balancing policies, and Kubernetes autoscaler thresholds. Therefore, while these metrics provide a practical proxy for system performance, they may not represent the entire spectrum of real-world service objectives.

**External validity:** Our experiments are based on Kubernetes, which is a widely used orchestration platform, but the results may not generalize directly to other orchestration frameworks or environments with different operational constraints. Furthermore, the tested workload patterns and injected failure scenarios represent a subset of real-world behaviors, and may not cover all production-grade conditions such as multi-region deployments, heterogeneous hardware, persistent storage failures, or complex cascading failures across dependent services. Network conditions in real deployments can also vary significantly, including higher jitter, packet loss, or bandwidth bottlenecks, which may change the effectiveness of our proposed method. Consequently, additional validation is required across larger-scale clusters and more diverse workloads to fully confirm generalizability.

**Conclusion validity and reliability:** Monitoring accuracy can impact the reported measurements. Prometheus-based instrumentation may suffer from sampling intervals, delayed scraping under high load, or missing data during unstable periods, potentially biasing reported values. We mitigate this by using consistent monitoring configurations across all runs and verifying metric completeness. Additionally, although we repeat experiments and report averaged results, statistical significance may still be influenced by the number of repetitions and the variance induced by the runtime environment. In future work, we plan to include stronger statistical testing (e.g., confidence intervals and hypothesis tests) and expand the scope of evaluation metrics to strengthen the conclusions.

### 4.3 Microservice resource efficiency

While prior evaluations demonstrate the consistent performance of our MCG approach, its scalability in large-scale cloud deployments warranted further evaluation. To address this need, we conducted empirical experiments under large-scale conditions to validate MCG operational efficacy. This subsection presents the findings from these scalability tests, providing quantitative evidence of MCG’s performance in realistic, resource-intensive environments. While prior analyses show that our MCG technique consistently performs well, more research was necessary to determine how scalable it is in large-scale cloud deployments. In particular, we launched more than 30 microservices by combining extra services from the *μ*Bench microservice benchmark with an extended version of the Bookinfo application. A Kubernetes cluster with 3 master and 7 worker nodes, each equipped with 4 virtual CPUs and 8 GB of RAM, was used to deploy the application.

**Fig 11:**
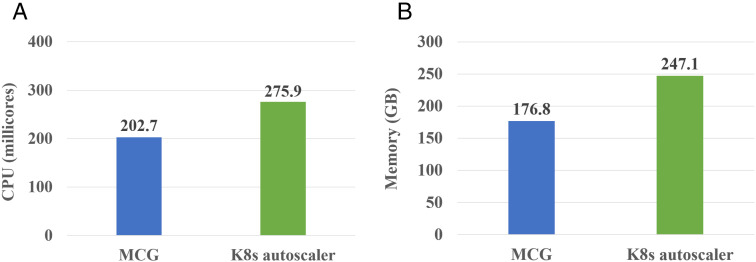
Computing resource usage comparison. **(a)** CPU usage. **(b)** Memory usage.

Using Locust tool, we generated 1000–1500 HTTP requests per second with different payload sizes (1–15 KB) to simulate high-concurrency workloads. High traffic surges, network latency variations, and dynamic scaling events were among the scenarios that were put to the test. Response time, request success rate, CPU and memory usage, and inter-node communication overhead were among the system characteristics we tracked. The results of these scalability tests are shown in Fig 11, offering quantifiable proof of MCG’s functionality in realistic, resource-intensive settings.

Fig 11 compares the computing resource utilization for Kubernetes’ autoscaler and the MCG scheme consumed for individual microservices in the Bookinfo application. The results demonstrate MCG ability to optimize resources, while maintaining strict latency SLOs. The MCG scheme reduced CPU usage by 26.5% (73,200 millicores) and memory consumption by 28.5% (70.3 GB) compared to Kubernetes’ autoscaling. This efficiency stems from MCG’s communication-aware scheduling, which dynamically prioritizes critical microservices, e.g., product and details services, while scaling down non-critical components. Additionally, these savings were achieved, while improving the system responsiveness. The MCG maintained a 95th-percentile latency of 160ms versus Kubernetes’ 180ms. The MCG scheme makes better scaling decisions by understanding service interactions in contrast to Kubernetes auto-scaling that considers basic performance metrics. Organizations benefit from reliable performance, lower expenses, and the ability to change scaling rules as necessary without suffering service outages.

## 5 Related work

Numerous researchers have been concerned about the difficulties associated with traffic scheduling in microservices-based platforms in recent years. In order to improve the performance of microservice applications under varying workloads, some research has focused on dynamically modifying service deployment and scaling during runtime. An important enabler in this field is the service mesh, which relieves application developers of underlying infrastructure details, so they can focus on business logic [3]. Several studies have explored traffic optimization for microservice applications, but a few address the unique challenges of service meshes. For example, Nautilus [18] focuses on microservice deployment in the cloud-edge continuum, considering public network latency. However, it overlooks resource optimization in local data centers, which is critical for efficient traffic steering. Similarly, OptTraffic [19] uses dynamic scheduling and local-first allocation to minimize cross-machine traffic and reduce end-to-end latency. Although effective, it does not fully account for the dynamic traffic patterns and heterogeneity inherent in the environments. A self-adaptive circuit breaker mechanism [20] is proposed that dynamically adjusts the open-state interval, i.e., the duration for which the circuit breaker remains in the open state before transitioning to half-open to probe service recovery. Unlike earlier circuit breaker optimization approaches that mainly tune static parameters such as failure thresholds, timeouts, or retry limits, its novelty is to treat the open-state interval itself as a runtime-adaptive parameter. By continuously adapting this waiting period based on observed system conditions, the approach improves request success rates and throughput, while reducing cascading failures in microservice deployments.

The problem of resource efficiency in cloud computing systems has been extensively studied in the literature [21–24]. These works focus on the co-scheduling of different applications, aiming at optimizing application performance. Enterprise resource planning system is different from these works. It seeks to reduce resource unbalance across different hosts so as to improve resource efficiency and provide end-to-end performance guarantees.

Graph partitioning has been used to optimize workload distribution and reduce communication costs in distributed systems. A container scheduling method based on graph partitioning is also proposed, in which applications are abstracted into a service communication graph to reduce inter-container communication overhead [25]. However, the approach does not consider the dynamic nature of microservice replicas, which is essential for load balancing in dynamic environments. Similarly, graph partitioning is applied to distributed stream processing by building a graph of communicating tasks and partitioning it based on runtime interactions [25]. Another notable effort is a service placement algorithm designed for hierarchical fog computing environments [26]. Before transferring compute-intensive services to cloud nodes, this algorithm preferably places them on fog nodes according to resource availability. Although it works well in fog environments, it does not address the unique difficulties faced by the service mesh like dynamic traffic flows and load balancing.

Existing graph partitioning approaches, such as MCGP [27], focus on optimizing task assignment for DAG-based workflows. Although being effective in minimizing data movement and improving workflow execution time, these methods do not account for service mesh features such as handling unmodified applications and inspecting HTTP headers. In a similar vein, a scheduler designed for stream processing systems optimizes job allocation through graph partitioning, but it ignores dynamic task communications and fails to fully reflect traffic patterns in heterogeneous clusters [28]. Our work addresses these limitations by proposing a graph-based scheme specifically designed for microservice environments. U By leveraging dynamic graph partitioning, our approach optimizes traffic flows, while considering heterogeneous resources and dynamic traffic patterns. This ensures efficient load balancing and reduces communication overhead for dynamically changing network conditions. Many research efforts were made to schedule containers of the same application or containers communicating frequently to the same or neighboring hosts in order to minimize cross-machine traffic [11,29–33]. In particular, CA-WFD [30] minimizes end-to-end latency and conserves bandwidth by distributing containers of the same application as far as possible across a single or adjacent machines. By grouping containers with high traffic into a container group and using the container group as a scheduling unit, Blender [33] makes traffic localization possible. By dynamically monitoring the traffic on each communication channel and moving containers to the same and nearby machines, NetMARKS [34] localizes service traffic. The fact that a microservice component typically has numerous replicas and that they use uniform traffic distribution is not taken into account in the aforementioned attempts. ON the other hand, our MCG provides traffic scheduling and local-first allocation capability to minimize server traffic and lower end-to-end latency.

Our method can be applied to containerized microservice platforms in general. Although their architectural organizations and APIs are different, other orchestration solutions like Docker Swarm, HashiCorp Nomad, and OpenShift offer comparable scheduling and scaling capabilities. Adjustments to service placement methods and interaction with their corresponding monitoring and scaling components can be necessary to adapt our approach to alternative platforms. However, our ideas of adaptive placement based on service communication patterns should remain applicable to the microservice orchestration platforms, indicating the possibility of wider use. To monitor, operate, and benchmark containerized cloud-native applications, our experimental evaluation makes use of a number of external technologies. Particularly, Locust replicates real-world workloads, Istio offers service mesh features like traffic routing and telemetry, and Prometheus is used to gather real-time performance data and events. In addition, resource allocation can be dynamically modified by the Kubernetes auto-scaler. Each of these techniques is essential for verifying the effectiveness and robustness of the suggested strategy.

The proposed approach has some drawbacks despite its advantages. Theoretically, it makes an assumption that service communication patterns are observable and rather stable, which might not be true in situations that are highly dynamic. Essentially, the solution depends on integrations and capabilities unique to the container orchestration platform, which may restrict porting to other distributed computing environments without alteration. Moreover, monitoring, tracking, and sustaining low-latency service communication may become increasingly challenging in large-scale implementations with diverse workloads. These concerns point to the directions for ouor future study to improve robustness and generalization.

## 6 Conclusion

This paper presents a novel architecture that makes use of service graph partitioning for microservice placement and traffic management. Unlike previous approaches, our proposal is designed to adjust in real-time to actual service graph topology, including communication patterns and traffic levels. Using collected telemetry data, it builds a communication graph for the services that enables us to use the minimal cut of the graph to pinpoint optimal partitions. This partitioned scheduling and traffic steering enhance application performance, while minimizing communication expenses between microservices. The traffic management capabilities of our system are dynamic, ensuring high availability, scalability, and resilience to failures among service replicas. Resultantly, it allows for better options for microservice placement by utilizing adaptive strategies that react to fluctuating network conditions.

We intend to expand our evaluation in subsequent work by putting the proposed approach to the test on a wider variety of microservice applications with various workloads and communication patterns. This will assist in evaluating the system’s resilience and generalizability over a range of application areas and deployment scales. It is also necessary to optimize the adaptive placement technique for large-scale and heterogeneous contexts and further investigate performance trade-offs.
